# Improving the productivity and profitability of maize (*Zea mays* L.) using optimum blended inorganic fertilization

**DOI:** 10.1515/biol-2022-0948

**Published:** 2024-09-09

**Authors:** Berhanu Bilate Daemo, Getahun Bore Wolancho, Zeleke Ashango

**Affiliations:** Department of Plant Science, Dawuro Tarcha Campus, Wolaita Sodo University, P.O. Box 138, Tarcha, Ethiopia

**Keywords:** grain yield, maize, NPSB rate

## Abstract

There is limited information on the best NPSB rate for maize production. Thus, the study aimed to determine the optimal NPSB fertilizer dose for maximizing maize yield and profitability. The nine treatments that included 0, 25, 50, 75, 100, 125, 150, 175, and 200 NPSB kg ha^−1^ were tested using a randomized complete block design with three replications. The results showed that increasing NPSB application significantly influenced maize plant height, ear height, hundred-seed weight, aboveground biomass yield, and grain yield. Specifically, the application of 150 kg ha⁻¹ NPSB consistently produced the tallest plants, highest ear heights, heaviest hundred-seed weights, and maximum aboveground biomass and grain yields across both sites. Economic analysis revealed that the 150 kg ha⁻¹ NPSB earned the highest net benefits and marginal rates of return, indicating its economic viability for smallholder farmers. Beyond 150 kg ha⁻¹, further increases in NPSB did not enhance yield or economic returns significantly. These findings underscore the importance of balanced fertilization for maximizing maize productivity and profitability while ensuring efficient resource use and environmental sustainability. Implementing optimized fertilizer practices with 150 kg ha⁻¹ NPSB can empower farmers in the study area and similar agroecological zones to achieve sustainable maize production and economic success.

## Graphical abstract

**Figure:**
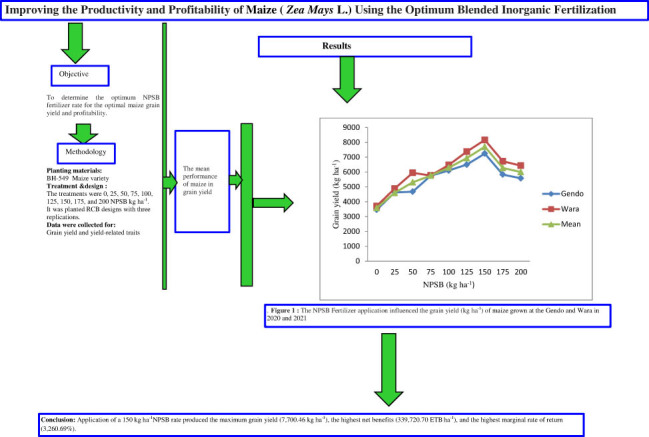

## 1 Introduction

Maize (*Zea mays* L.), a grass of the Poaceae family, is assumed to have originated in Mexico [1]. This versatile cereal crop holds global significance as a staple food, livestock feed, and industrial resource [2]. Its adaptability across climates bolsters food security and economic stability worldwide, with major cultivation in North America, Sub-Saharan Africa, and Latin America [2,3]. Maize cultivation reflects cultural significance in numerous societies [3]. Effective fertilization is critical for maximizing maize yields, enhancing soil fertility, and bolstering resilience to pests and climate change impacts [4]. Advanced fertilization techniques and sustainable farming practices have the potential to boost productivity, aiding global food security and fostering rural economic growth [4,5].

Maize was introduced to Ethiopia between the sixteenth and seventeenth centuries [6]. In Ethiopian agriculture, maize is the most widely cultivated cereal crop, ranking first in productivity and overall production while ranking second to teff in terms of area coverage [7,8]. About 9 million smallholder farmers in the 2020/2021 cropping season produced a total of 117,375,277 metric tons from an area of 3,419,008 ha [8]. Maize plays a crucial role in ensuring Ethiopia’s food security and economic growth for smallholder farmers [6]. Ethiopia faces significant and persistent food shortages due to its agriculture’s low productivity [7,9]. For instance, the current estimated average yield of maize on smallholder farmers’ farms is 3.3 t ha^−1^, which is significantly lower than the world average yield of 5.6 t ha^−1^ [2,8]. The main crop production challenges in sub-Saharan Africa include poor input utilization and inadequate soil fertility [10,11,12]. Furthermore, due to nutrient losses through soil erosion and other factors, along with a lack of inputs to restore soil fertility and uneven nutrient mining, tropical smallholder agricultural systems, including Ethiopia’s, are not sustainable [9,13,14].

The maintenance of soil fertility in sub-Saharan Africa, particularly in countries like Ethiopia, is challenged by rapid population growth, which hinders food production in smallholder farming [15]. Previous studies have emphasized the need to address declining soil fertility to enhance maize productivity through a balanced nutrient supply [16,17,18]. Inorganic fertilizers play a pivotal role in significantly boosting productivity and transforming unproductive soil [19,20]. They contribute to substantial increases in crop productivity, accounting for a significant portion of total production [21,22]. In addition, to meet growing demands, adopting improved crop varieties and chemical fertilizers is suggested [23].

Soil analysis data from Ethiopia revealed deficiencies in essential nutrients, including nitrogen (86%), phosphorus (99%), sulfur (92%), boron (65%), zinc (53%), and potassium (7%) [16,17,18]. In response, the Ministry of Agriculture in Ethiopia devised a blended multi-nutrient balanced fertilizer to rectify site-specific nutrient deficits and enhance crop production [17,24]. Currently, in the study area, a newly introduced blended NPSB 1The study technical term: NPSB means (18.9% N, 16.44% P, 6.95% S, and 0.1% B). fertilizer is being utilized by farmers with a blanket recommendation of 100 kg ha^−1^. It contains nutrients (18.9% N, 16.44% P, 6.95% S, and 0.1% B) substituting previously adopted NPS fertilizer [17,24]. However, the blanket recommended fertilizer rate of 100 kg ha^−1^ NPSB was developed elsewhere using different soil types, and it has not been proven to be the best dose for maize production in the study area to date. As a result, it is challenging for smallholder farmers and scholars to determine the appropriate dosage of NPSB fertilizers for maize production. Hence, better fertilizer application can enhance maize production more affordably and sustainably by adjusting to local climate, soil, and management practices [7,15,25]. Furthermore, Bekele et al. [26] have suggested a shift from blanket to site-specific fertilizer recommendations based on thorough soil and plant investigations to understand variations in crop response and profitability.

The International Maize and Wheat Improvement Center (CIMMYT) employs a systematic procedure for economic analysis to assess the cost-effectiveness of various agricultural interventions. This involves identifying innovations, collecting data on costs and benefits, analyzing direct and indirect costs, evaluating benefits such as yield increases, comparing costs and benefits, conducting sensitivity analyses, and communicating findings to stakeholders [27]. By following this method, CIMMYT aims to provide evidence-based insights into sustainable agricultural development and food security.

Thus, determining the optimal NPSB fertilizer dose for maize maximizes yield, resource efficiency, economic viability, environmental sustainability, and crop health in the study area. It ensures optimal growth, minimizes waste, maximizes profits, reduces pollution, and enhances plant resilience to pests and diseases. However, the study area lacked adequate information on the optimal NPSB fertilizer dose for maize production, necessitating tailored recommendations to enhance smallholder farmer productivity and profits. Therefore, the study aimed to determine the optimal NPSB fertilizer rate for maximizing maize grain yield and profitability in the study area and similar agroecological zones.

## 2 Materials and methods

### 2.1 Study area description

The fertilizer trial was conducted at Gendo and Wara locations from March to August in both the 2020 and 2021 cropping seasons. The Gendo trial site is located at 7°24′N and 37°38′E, with an elevation of 1,750 m.a.s.l. [28]. The area receives an average annual rainfall of 1,521 mm, with mean maximum and minimum temperatures of 24.5 and 15°C, respectively [28]. The Wara trial site is located at 7°34′N and 37°44′E, with an elevation of 1,550 m.a.s.l. [29]. The area receives an average annual rainfall of 1,400 mm, with mean maximum and minimum temperatures of 28.5°C and 16.5°C, respectively [29]. The detailed description of the soil characteristics of the study areas is presented in Table 1.

**Table 1:** Soil’s physical and chemical characteristics of the research sites before planting

Parameters	Unit	Gendo	Wara	Rating	Reference
Sand	%	36.0	16.0		
Clay	%	28.0	46.0		
Silt	%	36.0	38.0		
Textural class		Clay loam	clay		
pH	—	4.79	5.44	Strong acid (5.1–5.5)	[35]
CEC	Cmol(+)/kg soil	26.81	24.11	High (15–30)	[35]
P	mg/kg	4.94	6.73	Very low available (<15)	[35]
S	mg/kg	8.97	7.83	Very low (<10)	[35]
B	mg/kg	0.43	0.5	Deficiency (<0.5)	[35]
K	mg/kg	416.96	503.23	Optimum (190–600)	[35]
Total nitrogen	%	0.20	0.19	Low total nitrogen (<0.21)	[35]
Ca	mg/kg	2258.40	2581.4	High (2,000–4,000)	[35]
Mg	mg/kg	260.28	285.88	Moderate (120–360)	[35]

### 2.2 Sample preparation and soil analysis

Before planting, surface soil samples (0–30 cm depth) were collected from ten randomly chosen points zigzagging across Gendo and Wara trial sites using an auger. These samples were then combined into a single composite sample. From this composite, a 1.0 kg subsample was taken, which was subsequently air-dried, crushed, and sieved through a 2 mm mesh to ensure uniformity and preparation for detailed analysis of soil properties such as fertility, nutrient content, and texture. The soil texture was measured utilizing a Bouyoucos hydrometer [30], while the soil pH was determined using a digital pH meter on the supernatant suspension of a soil-to-water ratio of 1:2:5 [31]. Total nitrogen was analyzed by the Kjeldahl method as outlined by Bremner [32]. Exchangeable nutrients, including calcium (Ca), potassium (K), magnesium (Mg), sulfur (S), phosphorus (P), and boron (B), were estimated following the procedures described by Mehlich [33]. The cation exchange capacity (CEC) was determined using the ammonium acetate method by Chapman [34]. Table 1 presents the physical and chemical characteristics of the trial sites’ soil.

### 2.3 Experimental material, treatment, design of the experiment, and field management

A non-biofortified maize variety named BH 549 was used as a test crop. The BH 549 variety was selected due to its high yield, disease resistance, manageable height that simplifies harvesting, and adaptability. Furthermore, its uniform ear size and quality kernels enhance marketability. The farmer’s utilization rate of 100 kg ha^−1^ NPSB (18.9% N, 16.44% P, 6.95% S, and 0.1% B) was used as the basis for arranging the treatment. The treatments (nine levels of NPSB fertilizer) are presented in Table 2. Nine NPSB fertilizer levels were selected based on prior research, recommended rates from agricultural authorities, experimental design needs, nutrient requirements, practical constraints, and the exploratory nature of the study. This comprehensive approach was taken to capture a diverse range of nutrient application rates for maize production analysis. The trial was planted using a randomized complete block design with three replicates. Each experimental unit was 4.5 m wide × 4 m long, with blocks spaced 1 m apart. The plots within the blocks were spaced 0.5 m apart from each other. The maize seeds were sown at a plot size of 0.75 m with 0.3 m spacing between rows and plants, respectively. Plot sizes were chosen for meaningful treatment detection, considering equipment constraints, environmental variability, and following national research standards for maize fertilizer trial recommendations.

**Table 2:** Nine levels of blended NPSB fertilizers

Treatment code	NPSB fertilizer rate (kg ha^−1^)
T1	0
T2	25
T3	50
T4	75
T5	100
T6	125
T7	150
T8	175
T9	200

T: treatment.

The randomization process within each block was conducted systematically. The field was first divided into homogeneous blocks based on factors influencing maize growth. Random sequences were then generated for each block to ensure an equal chance of assignment for each treatment. Treatments were allocated to individual plots within each block according to the generated random sequence. This process was replicated across multiple plots within each block to enhance statistical power.

At planting time, the full NPSB was administered according to the rate for each plot. A total of 100 kg ha^−1^ of nitrogen fertilizer in the form of urea was applied twice: half at planting and the other half 45 days after planting. The timing and splitting of nitrogen fertilizer application were aligned with maize’s growth stages, optimizing nutrient uptake and reducing losses. Early growth was received nitrogen at planting, with subsequent applications timed for key growth phases. This approach could sustain optimal nitrogen levels, enhance plant health, and minimize environmental impact, which is crucial for maximizing maize productivity. Urea was chosen as the nitrogen fertilizer primarily due to its high nitrogen content and cost-effectiveness. Urea typically contains around 46% nitrogen, making it an efficient source of nitrogen for crop growth. Additionally, urea is readily available and relatively inexpensive compared to other nitrogen fertilizers, making it a practical choice for small- or large-scale agricultural applications. Moreover, urea is highly soluble in water, which facilitates its application through various irrigation systems.

The land was plowed three times before planting. The plowing frequency was aligned with local practices and soil conditions, aiming to create optimal seedbed conditions while minimizing soil disturbance and erosion risks. Weeding was performed three times (30, 45, and 60 days after planting) uniformly. Weeding schedules were based on the growth stage of the crop and the weed species present, with interventions timed to minimize competition for resources. Diseases and pests were monitored and controlled until the crops were harvested from the field. No farmyard manure, crop rotation with legumes, or crop residue retention was applied to the trial sites during or before this experiment.

### 2.4 Data collection

Plant and ear heights were measured using a tape measure for ten randomly selected plants from the central rows of each plot. The number of ears was counted from the central rows of ten randomly chosen plants per plot, and their mean was used for analysis. The grain yield per plot data was collected from the net plot area (12 m^2^). The grain yield (kg per plot) was measured by adjusting to a moisture content of 10% using a moisture tester and subsequently converted to kg ha^−1^ for analysis. The weight of a hundred seeds was sampled from each plot of cleaned seeds and counted using an electronic counter. This weight was then measured using a sensitive balance, with the seed’s moisture content corrected to 10%. The biomass yield was measured by selecting ten randomly chosen plants per plot from the middle rows at 90% physiological maturity. These plants were subsequently sun-dried in the field for 7 days until their weight stabilized. The biomass weight in kg per plot was then converted to kg ha^−1^ for analysis. Harvest index (%) was calculated using the formula HI = (weight of grain yield/(weight of grain yield + weight of Stover yield)) × 100.

### 2.5 Agronomic data and economic analysis

The study used SAS statistical software version 9.4 for variance analysis, and the traits that showed significant differences (*p* < 0.05) were further tested for mean separation using Duncan’s multiple range test (DMRT) [36]. Each location and season data was tested for homogeneity and normality using the Shapiro–Wilk w test and Bartlett’s test, and then the pooled analysis of variance was performed based on the generalized linear model (GLM) procedure for RCBD [37] as follows:

\[{Y}_{{ixjk}}=\mu +{T}_{i}+{S}_{x}+{L}_{j}+{{TS}}_{{ix}}+{{TL}}_{{ij}}+{{TSL}}_{{ixj}}+{R}_{k}+{{\mathrm{\varepsilon }}}_{{ixjk}},]\]

where \[{Y}_{{ixjk}}]\] is the observed value of treatment \[i]\] in replication \[k]\] of season *x* and location \[j]\] , *μ* is the grand mean of the trait, \[{T}_{i}]\] is the effect of treatment *i*, \[{S}_{x}]\] is the effect of season *x*, \[{L}_{j}]\] is the effect of location \[j]\] , \[{{TS}}_{{ix}}]\] is the interaction effect of treatment \[i]\] with season *x*, \[{{TL}}_{{ij}}]\] is the interaction effect of treatment \[i]\] with location *j*, \[{{TSL}}_{{ixj}}]\] is the interaction effect of treatment \[i]\] with season *x* and location *j*, \[{R}_{k}]\] is the effect of replication \[k]\] , and \[{\varepsilon }_{{ixjk}}]\] is the error (residual) effect of treatment \[i]\] in replication *k* of season *x* and location \[j]\] .

The economic analysis was calculated for each treatment to consolidate the statistical analysis of the agronomic data. The economic analysis was estimated based on the overall grain yield mean across locations and seasons, calculated using the total variable costs (TVCs) and net benefits (NBs) of each treatment. In the present study, the cost of NPSB chemical fertilizer and labor costs for its application varied, while other costs were kept constant for each treatment. This approach allowed for a focused comparison of the economic impact of different NPSB fertilizer rates on maize production. According to the CIMMYT [27] procedure, farmers would achieve yields 10% lower than the obtained yield in the experiment, and then the mean maize grain yield was adjusted in the economic analysis by subtracting 10% from the actual yield. Economic evaluations were computed for the TVC, gross field benefit (GFB), NB, and marginal rate of return (MRR) ratios using the method described by CIMMYT [27].

The TVC was calculated by combining all variable costs, including chemical fertilizer and labor costs, while keeping other costs constant for each treatment. The cost of NPSB fertilizer was 42.20 ETB kg^−1^ and the cost of application of NPSB fertilizer was 500.00 ETB ha^−1^.

The GFB was derived by multiplying the adjusted total grain yield (kg ha^−1^) for each treatment by the current open price of kg per Ethiopian birr (50.00 ETB kg^−1^) for maize.

The NB was obtained by (GFB − TVC).

The MRR% was computed by \[{\mathrm{MRR}}( \% )=\frac{\triangle {\mathrm{NB}}}{\triangle {\mathrm{TVC}}}{\mathrm{\times }}100]\] where \[\triangle {\mathrm{NB}}]\] was the change in the NB and \[\triangle {\mathrm{TVC}}]\] was the change in TVC between any pair of treatments.

## 3 Results and discussion

### 3.1 Plant and ear height

Applying varying amounts of NPSB fertilizer had a substantial effect (*p* ≤ 0.01) on the plant and ear height of maize (Table 3). The mean results from two seasons revealed that at Gendo, the tallest plants, reaching 2.69 m, were recorded with the application of 150 kg ha⁻¹ NPSB. This was followed by plants measuring 2.37 m with a 200 kg ha⁻¹ NPSB application. In contrast, the shortest plants, measuring 1.85 m, were found in the unfertilized plot (Table 4). Similarly, at Wara, the tallest plants, reaching 2.84 m, were achieved with the application of 150 kg ha⁻¹ NPSB, followed by plants measuring 2.75 m with a 175 kg ha⁻¹ NPSB rate. The shortest plants at Wara, measuring 1.90 m, were also observed in the unfertilized plot (Table 4). Regarding ear height, a similar trend was evident. At Gendo, the maximum ear height (1.23 m) was achieved with a 150 kg ha⁻¹ NPSB rate, while the minimum ear height (0.88 m) was observed in the unfertilized plot (Table 4). At Wara, the highest ear height (1.34 m) was attained with a 150 kg ha⁻¹ NPSB rate, followed by 1.36 m with a 175 kg ha⁻¹ NPSB rate, which was statistically equivalent. Overall, the mean performance across locations showed that a 150 kg ha⁻¹ NPSB rate resulted in the highest plant height (2.77 m) and ear height (1.30 m) (Table 4).

**Table 3:** Pooled analysis of variance of locations over seasons for plant and ear height, number of ears per plant, hundred-seed weight, biomass yield, harvest index, and grain yield of maize

Source of variations		Mean squares						
	DF	PH	EH	NE	HSW	BY	HI	GY
Treatment (T)	8	0.72**	0.10**	0.06^NS^	168.49**	152,617,030**	13.01^NS^	17467535.8**
Location (L)	1	1.26**	1.33**	0.001^NS^	0.23^NS^	20,106,839**	108.18*	10845604.6**
Year (Y)	1	0.05^NS^	0.44**	0.01^NS^	3.34^NS^	5,967,670^NS^	90.62*	4846505.2**
Replication	2	0.002	0.004	0.05	10.34	24,097,776	157.07	449541.2
T × L	8	0.03^NS^	0.009^NS^	0.01^NS^	17.87^NS^	835,928^NS^	6.69^NS^	525270.5^NS^
T × Y	8	0.02^NS^	0.008^NS^	0.02^NS^	5.74^NS^	577986^NS^	13.02^NS^	377567.5^NS^
T × Y × L	8	0.01^NS^	0.007^NS^	0.01^NS^	4.21^NS^	733,049^NS^	9.11^NS^	552827.5^NS^
Residual	70	0.01	0.004	0.012	8.97	1,583,987	6.54	377164.6

Key: NS, *, **, = non-significant at 0.05, significant at 0.05, and highly significant at 0.01 level of probability, respectively, PH = plant height (m), EH = ear height (m), NE = number of ears per plant, HSW = hundred-seed weight, BY = aboveground dry biomass yield (kg ha^−1^), HI = harvest index (%) and GY = grain yield (kg ha^−1^).

**Table 4:** Influence of NPSB rates on the mean performance of plant and ear heights of maize grown at Gendo and Wara

NPSB (kg ha^−1^)	Plant height (m)			Ear height (m)		
	Gendo	Wara	Mean	Gendo	Wara	Mean
0	1.85^e^	1.90^d^	1.88^d^	0.88^d^	1.08^d^	0.99^d^
25	2.21^cd^	2.38^c^	2.30^c^	0.95^cd^	1.13^d^	1.04^d^
50	2.23^bcd^	2.39^c^	2.31^c^	1.06^abcd^	1.25^c^	1.16^c^
75	2.17^d^	2.39^c^	2.28^c^	1.03^bcd^	1.26^c^	1.50^c^
100	2.28^bcd^	2.47^c^	2.37^c^	1.07^abc^	1.30^bc^	1.18^bc^
125	2.32^bcd^	2.65^b^	2.48^b^	1.08^abc^	1.31^abc^	1.20^bc^
150	2.69^a^	2.84^a^	2.77^a^	1.23^a^	1.37^a^	1.30^a^
175	2.36^bc^	2.72^ab^	2.54^b^	1.16^ab^	1.36^ab^	1.26^ab^
200	2.37^b^	2.70^b^	2.53^b^	1.12^abc^	1.31^abc^	1.21^bc^
LSD (0.05)	0.16	0.12	0.09	0.19	0.06	0.08
CV (%)	11.17	10.6	10.92	7.37	7.10	7.5

Mean values within the same column followed by the same letter or no letters are not significantly different.

These data indicate that increasing the NPSB fertilizer rate from 0 to 150 kg ha⁻¹ significantly enhanced both plant and ear height traits; however, beyond this point, additional NPSB fertilizer does not further increase these traits. The observed increase in plant and ear height with higher NPSB rates can be attributed to the improved nutrient availability, which promotes cell elongation and overall plant growth. The findings suggest that applying NPSB fertilizer optimizes the physiological development of maize, leading to taller plants and higher ear placement, which are desirable traits for improving light capture and potentially increasing grain yield. However, the lack of response in plant height beyond the 150 kg ha⁻¹ NPSB rate indicates a threshold beyond which additional fertilizer does not provide further benefits. This could be due to the plants reaching their maximum genetic potential for height or the soil’s nutrient saturation point. Therefore, for optimal growth and resource use efficiency, a 150 kg ha⁻¹ NPSB rate is recommended for maize cultivation in the study area. Implementing these findings can help farmers achieve better plant growth, leading to improved crop management practices and potentially higher yields. Understanding the optimal fertilizer rate also ensures that resources are used efficiently, avoiding unnecessary expenditure on excess fertilizer and minimizing environmental impact. This finding is in agreement with Mekuria et al. [18], Tekulu et al. [38], and Abera and Adinew [39], who concluded that the application of NPSB fertilizer at a higher rate would significantly increase maize plant height and ear height compared to the unfertilized plot.

### 3.2 Ear number, harvest index, and hundred-seed weight

The ear number of the plant and harvest index showed non-significant results (*p* ≤ 0.05) for applying NPSB fertilizer, while the hundred-seed weight revealed significant differences among the treatments (*p* ≤ 0.01) (Table 3). At Gendo, the application of a 150 kg ha^−1^ NPSB rate resulted in the highest hundred-seed weight of 38.0 g, closely followed by weights of 37.33 and 35.84 g achieved with 125 and 175 kg ha^−1^ NPSB rates, respectively (Table 5). Similarly, at the Wara location, the maximum hundred-seed weight of 39.66 g was recorded with a 150 kg ha^−1^ NPSB rate, while the lowest weight of 24.33 g was observed in the unfertilized plot.

**Table 5:** Influence of NPSB fertilizer rates on the mean performance of ear number per plant, hundred-seed weight (g), and harvest index (%) of maize grown at the Gendo and Wara

NPSB (kg ha^−1^)	Ear number per plant			Hundred-seed weight (g)			Harvest index (%)		
	Gendo	Wara	Mean	Gendo	Wara	Mean	Gendo	Wara	Mean
0	1.0	1.0	1.0	28.16^d^	24.33^e^	26.25^e^	29.57	29.97	29.77
25	1.05	1.0	1.03	32.66^c^	29.33^d^	31.00^d^	30.25	31.87	32.30
50	1.0	1.0	1.0	34.33^bc^	36.66^abc^	35.50^bc^	30.25	35.27	32.76
75	1.05	1.11	1.08	35.16^abc^	34.33^c^	34.70^c^	32.45	32.49	32.48
100	1.11	1.22	1.17	34.66^abc^	36.00^bc^	35.33^c^	30.02	31.31	30.66
125	1.09	1.0	1.05	37.33^ab^	37.83^ab^	37.58^ab^	31.28	33.65	32.47
150	1.0	1.0	1.0	38.00^a^	39.66^a^	38.83^a^	31.17	33.71	32.44
175	1.16	1.22	1.19	35.84^abc^	35.83^bc^	35.83^bc^	29.78	32.32	31.06
200	1.10	1.0	1.05	33.83^bc^	36.83^abc^	35.33^bc^	29.51	32.20	30.85
LSD (0.05)	0.11	0.13	.0.12	3.51	3.49	2.43	3.04	4.50	2.08
CV (%)	9.95	11.0	10.21	3.51	8.63	8.68	8.75	7.01	8.11

Mean values within the same column followed by the same letter or no letters are not significantly different.

The overall mean separation revealed that the highest hundred-seed weight, 38.83 g, was attained with a 150 kg ha^−1^ NPSB application, closely followed by 37.58 g with a 125 kg ha^−1^ NPSB application, which was statistically similar (Table 5). These findings indicate a significant increase in hundred-seed weights with increasing NPSB rates up to 150 kg ha^−1^. Beyond this rate, further increments in NPSB do not substantially enhance the seed weight, suggesting an optimal fertilizer application threshold for maximizing seed weight.

The increase in hundred-seed weight with higher NPSB rates can be attributed to the enhanced availability of essential nutrients, which support seed development and maturation. Nitrogen, phosphorus, sulfur, and boron play critical roles in various physiological and biochemical processes within the plant, leading to improved seed filling and overall seed quality. This improved nutrient uptake likely facilitates better energy storage and structural development within the seeds, resulting in heavier and more robust seeds. Therefore, optimizing NPSB fertilizer application is crucial for achieving maximum seed weight, which is a key determinant of maize yield and quality. This finding aligns with previous studies by Chinasho et al. [40] and Abebe et al. [41], which reported significant differences in hundred-seed weights when applying various levels of NPSB fertilizer. These studies suggest that the application of NPSB fertilizer not only enhances overall plant growth but also positively impacts seed development and quality.

### 3.3 Aboveground biomass yield

The aboveground biomass yield was significantly influenced by applying NPSB fertilizer (Table 3). At Gendo, the highest aboveground dry biomass yield was achieved with a 150 kg ha⁻¹ NPSB rate, producing 23,337 kg ha⁻¹ (Table 6). This was followed by yields of 20,723 and 20,330 kg ha⁻¹ with the 125 and 100 kg ha⁻¹ rates, respectively. Similarly, at Wara, the 150 kg ha⁻¹ NPSB rate resulted in the highest yield of 24,297 kg ha⁻¹. The overall mean performance across both locations showed that the 150 kg ha⁻¹ NPSB rate produced the maximum aboveground dry biomass yield of 23,817 kg ha⁻¹, with the 125 kg ha⁻¹ rate yielding 21,310 kg ha⁻¹ (Table 6).

**Table 6:** NPSB fertilizer application influenced the mean performance of aboveground dry biomass yield (kg ha⁻¹) and grain yield (kg ha⁻¹) of maize grown at Gendo and Wara

NPSB (kg ha^−1^)	Biomass yield (kg ha^−1^)			Grain yield (kg ha^−1^)		
	Gendo	Wara	Mean	Gendo	Wara	Mean
0	11,620^f^	12,396^f^	12,008^h^	3455.56^e^	3704.55^g^	3580.06^g^
25	15,075^e^	15,359^e^	15,217^g^	4627.78^d^	4883.33^f^	4597.56^f^
50	15,565^e^	17,072^d^	16,318^f^	4673.78^d^	5945.45^de^	5309.62^e^
75	17,732^d^	17,781^d^	17,756^e^	5747.68^c^	5763.64^e^	5755.55^e^
100	20,330^b^	20,693^bc^	20,611^bc^	6103.03^bc^	6471.27^cd^	6290.15^c^
125	20,723^b^	21,896^b^	21,310^b^	6496.23^ab^	7369.70^b^	6932.96^b^
150	23,337^a^	24,297^a^	23,817^a^	7242.59^a^	8158.33^a^	7700.46^a^
175	19,384^bc^	20,819^bc^	20,102^cd^	5825.66^bc^	6722.73^c^	6274.19^c^
200	18,749^cd^	19,970^c^	19,360^d^	5577.63^c^	6428.03^cd^	6002.33^cd^
LSD(0.05)	1406.7	1539.2	1024.8	747.09	636.27	500.05
CV (%)	9.84	8.5	7.80	11.51	9.02	10.51

Mean having the same letter(s) for a trait indicates that there is no significant difference.

These results indicate that increasing the NPSB application rate leads to higher biomass yields, with the 150 kg ha⁻¹ rate being the most effective and consistent across different conditions. The data demonstrate that increasing the NPSB fertilizer rate from 0 to 150 kg ha⁻¹ significantly increases the aboveground biomass yield. The increase in biomass yield could be due to improved dry matter production resulting from the optimal application of NPSB fertilizer, promoting vigorous and healthy plant growth.

Nitrogen, phosphorus, sulfur, and boron are essential for various physiological processes, including photosynthesis, protein synthesis, and cell division. The higher nutrient availability at the 150 kg ha⁻¹ rate likely promotes better overall plant development, leading to greater biomass accumulation. Therefore, applying a 150 kg ha⁻¹ NPSB rate is recommended for maximizing aboveground biomass yield, which is crucial for both the economic and agronomic success of crop production. These findings are consistent with the research by Adugna et al. [24], Tekulu et al. [38], Belay and Adare [42], and Tadesse and Sultan [43], who also reported significant increases in aboveground dry biomass yield with increasing NPSB rates up to an optimal level. The correlation between NPSB application and biomass yield underscores the critical role of balanced fertilization in maximizing crop productivity.

### 3.4 Grain yield

The dose of NPSB fertilizer had a substantial effect (*p* < 0.001) on the maize grain yield (Table 3). Applying a 150 kg ha⁻¹ NPSB rate consistently resulted in the highest grain yields at both Gendo and Wara locations (Table 6). At Gendo, this rate produced a maximum grain yield of 7242.59 kg ha⁻¹, significantly higher than the 6496.23 kg ha⁻¹ yield from the 125 kg ha⁻¹ rate and more than double the 3455.56 kg ha⁻¹ yield from the unfertilized plot. Similarly, at Wara, the 150 kg ha⁻¹ NPSB rate achieved the highest grain yield of 8158.33 kg ha⁻¹, with the unfertilized plot yielding the lowest at 3704.55 kg ha⁻¹. The overall mean performance across both locations showed the 150 kg ha⁻¹ NPSB rate producing the highest average grain yield of 7700.46 kg ha⁻¹, followed by the 125 kg ha⁻¹ rate with 6916.30 kg ha⁻¹ (Table 6).

These results demonstrate the substantial impact of NPSB fertilizer on the grain yield, with the 150 kg ha⁻¹ rate being the most effective in maximizing production. The unfertilized plots consistently produced the lowest, highlighting the critical role of fertilization. Therefore, while applying 150 kg ha⁻¹ NPSB is optimal for maximizing grain yield, it is also essential to consider the cost and resource implications of different fertilization rates to achieve the best economic outcome.

In comparison to the blanket recommended NPSB rate, the application of a 150 kg ha⁻¹ NPSB rate increased the maize grain yield by 19 and 26.1% at Gendo and Wara, respectively. Additionally, the findings showed that increasing NPSB rates from 0 to 150 kg ha⁻¹ increased the grain yield of maize by 109.6 and 120.2% over the unfertilized plots in Gendo and Wara, respectively. The observed increase in maize grain yield could be due to the plant growing larger and healthier when applying the optimum level of fertilizer, as well as the enhanced positive interaction between nutrients in the blended fertilizer.

However, increasing the NPSB rate from 150 to 200 kg ha⁻¹ did not increase the maize grain yield in either location. This could be due to a maximum efficiency point at which an additional kg of fertilizer does not produce sufficient extra grain yield to justify its cost, thus the recommendation should focus on maximizing profitability. Therefore, the 150 kg ha⁻¹ NPSB fertilizer rate appears to be the optimal requirement for maximizing maize crop yield in the study area and similar agroecological zones, balancing high productivity with economic efficiency.

These findings are in line with previous studies by Mengistu [6], Sigaye et al. [9], Tekulu et al. [38], Tunebo et al. [44], and Orebo et al. [45], who conducted fertilizer trials in different soil types at various levels of fertilizer for maximum grain yield, concluding that maize grain yield increased as the fertilizer level increased up to an optimum level, and then there was no further increment in maize grain yield. The correlation between NPSB application and grain yield highlights the critical role of balanced fertilization in maximizing crop productivity.

### 3.5 Economic analysis

The economic analysis detailed in Table 7 presents a comprehensive evaluation of the TVCs, GFB, NBs, and MRR for maize production. The economic analysis of maize production using NPSB fertilizer demonstrated significant benefits over unfertilized plots in terms of both grain yield and NBs. By focusing on the variable costs associated with NPSB fertilizer application while keeping other costs constant, the study provided a clear comparison of different fertilizer rates. The results showed that applying 150 kg ha⁻¹ of NPSB fertilizer obtained the highest NB of 339720.70 ETB per hectare, highlighting the substantial economic advantage of this treatment. The second-highest NB was recorded with a 125 kg ha⁻¹ rate, amounting to 306233.2 ETB per hectare.

**Table 7:** Partial budget analysis for NPSB fertilizer rate based on the grain yield of maize grown at Gendo and Wara

NPSB (kg ha^−1^)	UGY (kg ha^−1^)	AGY (kg ha^−1^)	Fertilizer cost (ETB ha^−1^)	Fer. app. cost (ETB ha^−1^)	TVC (ETB ha^−1^)	GFB (ETB ha^−1^)	NB (ETB ha^−1^)	MRR (%)
0	3580.06	3222.05	0.0	0.0	0.0	161102.70	161102.7	—
25	4597.56	4137.80	1050.0	500.0	1550.0	206890.20	205340.2	2854.03
50	5309.62	4778.66	2100.0	500.0	2600.0	238932.90	236332.9	2951.69
75	5755.55	5180.00	3150.0	500.0	3650.0	258999.75	255349.75	1811.13
100	6290.15	5661.14	4200.0	500.0	4700.0	283056.75	278356.75	2191.14
125	6932.96	6239.66	5250.0	500.0	5750.0	311983.20	306233.2	2654.90
150	7700.46	6930.41	6300.0	500.0	6800.0	346520.70	339720.7	3189.29
175	6274.19	5646.77	7350.0	500.0	7850.0	282338.55	274488.55	D
200	6002.33	5402.10	8400.0	500.0	8900.0	270104.85	261204.85	D

Note: 1US Dollar = 55 ETB current exchange rate.

Key: UGY = unadjusted grain yield kg ha^−1^, AGY = 10% adjusted grain yield kg ha^−1^, TVC = total variable costs (ETB ha^−1^), GFB = gross field benefit (ETB ha^−1^), NB = net benefit (ETB ha^−1^), MRR = marginal rate of return (%), and D = dominated.

The MRR further emphasized the efficiency of these treatments. The 150 kg ha⁻¹ rate achieved an MRR of 3189.29%, meaning each 1 ETB invested returned 31.89 ETB, whereas the 125 kg ha⁻¹ rate had an MRR of 2654.90%, obtaining 26.55 ETB for each 1 ETB invested. These exceptionally high MRR values indicate that both fertilizer rates are not only profitable but also highly efficient investments, making them attractive options for farmers seeking to maximize their economic returns. Hence, small-scale farmers are encouraged to use a 150 kg ha⁻¹ NPSB (nitrogen, phosphorus, sulfur, and boron) fertilizer rate to increase maize profitability, as this rate has been identified through research as optimal for maize growth and yield in the study area and other regions with similar soil conditions. This recommendation ensures that crops receive balanced nutrition, leading to healthier plants and higher yields, thereby increasing profitability. Additionally, it promotes economic efficiency by preventing both over-fertilization and under-fertilization, which can be costly and detrimental to crop performance. By adopting this practice, farmers can achieve better yields, enhance their income, and contribute to the sustainable management of agricultural resources.

## 4 Conclusion

In conclusion, this study identified the optimal NPSB fertilizer dose for maize cultivation in the study area, emphasizing its critical role in maximizing yield, resource efficiency, economic viability, environmental sustainability, and crop health. The study recommends applying 150 kg ha⁻¹ of NPSB fertilizer to achieve superior maize growth and grain yield, supported by significant improvements in the plant height, ear height, hundred-seed weight, aboveground biomass yield, and grain yield. Economic analysis revealed that the 150 kg ha⁻¹ NPSB rate earned the highest NBs (339720.70 ETB per hectare) and MRR (3189.29%), indicating its economic viability for smallholder farmers. Beyond this optimal rate, further increases in fertilizer dosage do not significantly enhance crop performance, indicating a balance between maximizing productivity and economic efficiency. These findings provide valuable insights for smallholder farmers, offering tailored recommendations to enhance productivity, profitability, and sustainable agricultural practices in similar agroecological zones.
